# Modelling of What‐Where‐When Everyday Memories in Rats

**DOI:** 10.1111/ejn.70278

**Published:** 2025-10-14

**Authors:** Kayleigh Kanakis, Richard G. M. Morris, Francesco Gobbo

**Affiliations:** ^1^ School of Psychology and Neuroscience, College of Medical, Veterinary, and Life Sciences University of Glasgow Glasgow UK; ^2^ Centre for Discovery Brain Sciences University of Edinburgh Edinburgh UK; ^3^ UK Dementia Research Institute University of Edinburgh Edinburgh UK

## Abstract

Episodic memories contain information about the nature of an event, the place where it happened and the time when it occurred. In animals, the term ‘episodic‐like memory’ is preferred to refer to mnemonic instances containing these three features, commonly referred to as ‘what‐where‐when’. Models to study episodic‐like memory have been proposed in corvidae and rodents, although their use in neuroscience research has been limited due to certain limitations and potential ambiguities. Although the neurological correlates of ‘what‐where‐when’ have been identified in neuronal types such as place and time cells, it is unclear how they contribute to form a unitary representation or how this information can be accessed during memory recall, either holistically or differentially. Here, we outline two new behavioural paradigms based on the everyday memory task that we have developed to model what and when components as well as ‘where’ information. In Experiment 1 (E1), we demonstrate that rats are able to learn two distinct food positions on a daily basis and retrieve them independently. In E2, we establish that rats can learn that two flavours are replenished at different times after an initial sampling, thus using the temporal component to guide their decision making. These two tasks can therefore provide the basis to study how the item, location and time information of a memory are stored and accessed by the brain. This should be observable in single‐unit recording or calcium‐imaging studies.

## Graphical abstract

Animal models have greatly advanced our understanding of learning and memory. However, reliable protocols recapitulating aspects of episodic‐like memory have been difficult to implement. In this study, we developed two behavioural paradigms for rats that explicitly model what and where information (Experiment 1) or where and when (Experiment 2). Both experiments are based on the use of flavoured rewards hidden in a two‐dimensional arena. These protocols will support the research on the formation of everyday memories and the access of the stored information during recall and decision making.

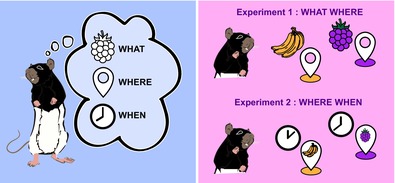

**Abbreviations**
ANOVA: one‐way analysis of variance
B: banana
C: chocolate
CT: choice trial
CT 3 h: choice trial at 3 h
CT 10 min: choice trial at 10 min
DG: dentate gyrus
E: east
FP: food preference
MM: marshmallow
PC: piña colada
PI: performance index
PT: probe trial
S: south
ST: sample trial
SW: Sandwell
Vb: very berry
W: west

## 1 Introduction

Among the different forms of memory, episodic memory is perhaps the closest to our everyday concept of remembering (Squire 1984). When it comes to human experience, resurrecting such mental representations is often described as ‘mental time travel’ (Tulving 1985). At least a subset of these recall events involves the recollection of a unique ‘event’ that happened once. These are often re‐lived in the first person, with the added awareness of it being a recollection, or autonoesis (Tulving 2002). These memories of past experiences contain information about *what* happened, *where* and *when* it happened, plus a number of pieces of information such as context and temporal sequences (Nyberg et al. 1996). The concept of episodic memory has been extended to ‘episodic‐like memory’ to include its behavioural manifestation in nonhuman animals, as it is debated and fundamentally unknown whether animal experience can involve an analogous sense of self (Tulving 2005; McKenzie et al. 2024). It is generally accepted that hippocampal function is associated with episodic memory and, by extension, episodic‐like memory (Allen and Fortin 2013), as demonstrated by studies in amnesic patients such as H.M. (Scoville and Milner 1957).

Episodic‐like facets of memory have been demonstrated in primates (Gaffan 1974; Schwartz et al. 2002), birds (Clayton and Dickinson 1998), rodents (Babb and Crystal 2006a) and even invertebrates (Jozet‐Alves et al. 2013). These and other studies have demonstrated that animals can learn and use *what‐where*‐*when* information in behavioural paradigms, often involving the search for various types of food. Evidence for nonlocal or out‐of‐context recall, translating the concept of mental time travel without the requirement of autonoesis, has come from both behavioural and neural recording experiments (Ježek et al. 2010, 2011; Kay et al. 2020; Gobbo et al. 2022). This has often been associated with a role for replay in decision‐making, notwithstanding the ongoing debate on the relative contribution of replay to planning and memory consolidation (Ólafsdóttir et al. 2018; Comrie et al. 2022). Conversely, single experience encoding has been addressed in birds (Clayton and Dickinson 1998) and rodents (Takeuchi et al. 2016; Wagatsuma et al. 2017; Tse et al. 2023). Like flashbulb memories, the formation of these memories is often associated with novel or unexpected events (Hirst et al. 2009). However, even in humans, recall precision is often questionable, and even substantial aspects can be misremembered with absolute confidence in their veracity (Hirst et al. 2015). Hence, time travel often, if not always, relies on scene reconstructive activity operated by the hippocampal formation (Hassabis and Maguire 2007). It must be noted that, in the study of episodic memory, not all aspects need to be present at once. For instance, retelling a story requires mental time travel, whereas recognising images or remembering word lists do not necessarily. The possibility of doing that is often regarded as sufficient for them to measure episodic memory (Collaro et al. 2024).

In rodents, episodic‐like memory is sometimes modelled with novel object recognition paradigms and variants thereof. However, the simplest of such protocols can involve the hippocampus but does not depend on it (Ennaceur and Delacour 1988; Langston and Wood 2010). Tasks of higher complexity, such as object‐in‐place, have been demonstrated to be hippocampal‐dependent; typically, they involve the assessment of multiple information by animals, such as *what*, *where* and *which* (Easton and Eacott 2010; Aggleton and Nelson 2020). For instance, Eacott and Norman (2004) showed that hippocampal lesions impair discrimination of ‘object‐context‐in place’ tasks. Babb and Crystal (2006a) have used food rewards in particular positions of a radial arm maze to explicitly model *what‐where* associations and explored their sensitivity to devaluation. The same setup has also been extended to *what‐where‐when* associations (Roberts et al. 2008; Zhou and Crystal 2009) using a depletion‐replenishment paradigm, also used by Clayton et al. (2001) in scrub‐jays, which currently constitutes the benchmark for episodic‐like tests in animals.

A limitation of these tasks is the lack of a clear separation between (a) decision making and (b) on‐line execution of the trial. This makes them unsuitable to study the neural representations associated with planning and remembering, as bona fide planning events may just happen at unpredictable decision points. As discussed above, several studies in purely spatial tasks have provided evidence of task‐related nonlocal reactivation, as well as replay of past, correct and alternative directions (Ainge et al. 2007; Gillespie et al. 2021; Gobbo et al. 2022). Among them, the event arena paradigm, where animals learn to retrieve food in a specific location in a two‐dimensional arena, offers such a possibility, as animals enter the arena from designated extra‐arena start boxes, where rats make their decisions (Bast et al. 2005; Gobbo et al. 2022).

On the other hand, recency protocols using navigation in watermazes and arenas are often considered episodic‐like in nature and sensitive to hippocampal inactivation (Steele and Morris 1999; Bast et al. 2005). One caveat is that most protocols developed in these apparatus use multiple trials to learn the position of the reward and therefore do not strictly meet the criterion of single‐trial encoding—although single‐trial learning has been demonstrated in a number of studies (Wang et al. 2010; Takeuchi et al. 2016; Nonaka et al. 2017; Rossato et al. 2018). In these studies, the main difference between single‐trial and multiple‐trial learning seems to be the strength of the memory (Wang et al. 2010) or encouraging the use of particular strategies (e.g., allocentric navigation); however, to avoid confusion, we will adopt the term ‘everyday memory’ for the latter. In both cases, the main limitation of protocols developed so far has been their conflation of episodic‐like or everyday memory with spatial memory. In fact, behavioural tasks such as the event arena only model the *where* component. Although variants have been developed to model different navigation strategies (Broadbent et al. 2020) and contextual information (Prodan et al. 2022), a version of the task incorporating explicitly *what* and *when* information is currently missing.

Recordings from the hippocampus and associated areas have found evidence for possible neural correlates of the various types of information, such as place cells (O'Keefe 1976), grid cells (Fyhn et al. 2004), item‐position cells (Komorowski et al. 2009) and time cells (Eichenbaum 2014). With the—partial—exception of spatial information, their involvement in the representation of episodic memories is still unexplored. For instance, to what extent nonspatial information is represented in the hippocampus, and how these different aspects interact with each other is still largely unknown (McKenzie et al. 2016; O'Keefe and Krupic 2021). Similarly, it is still debated how and to what extent the replay of spatial information is used to make memory‐based decisions, and virtually nothing is known about the other modalities (Ólafsdóttir et al. 2018).

To date, no behavioural protocol has been developed in rats that (i) allows us to monitor all three *what*, *where* and *when* aspects of episodic memory and (ii) provides a reasonable separation between task execution and planning. Variants of the event arena task where animals would distinguish between separate goals (*what*) or replenishment time (*when*) would provide a behavioural protocol to observe if, and how, memory‐based complex information is reactivated in an anticipatory way (Kay et al. 2020; Gobbo et al. 2022). Here, we sought to close this gap by developing modified protocols in the everyday arena to model episodic‐like *what*, *where* and *when* information based on an allocentric definition of coordinates and used different flavours of food to model *what* (Day et al. 2003; Tse et al. 2007).

## 2 Materials and Methods

### 2.1 Animals

Twelve young male Lister‐Hooded rats were used in this experiment. They were purchased from Charles River Laboratories (Currie, United Kingdom) and housed two or four per cage. At the start of the experiment, the rats were 2.5 months old and had a mean weight of 285.67 g. Rats were maintained in a 12‐h light/12‐h dark cycle. For the first week, water and food were available ad libitum. The animals were then food restricted and maintained at 85%–90% of their ad libitum weight (being fed around 22 g of food per day per animal). Experimental procedures in this study were performed strictly in line with the Animals (Scientific Procedures) Act 1986 in compliance with British Law and regulations and the European Communities Council Directive of 24 November 1986 (86/609/EEC) legislation.

### 2.2 Apparatus and Materials

The everyday apparatus is a 1.5 × 1.5 m arena, comprising a 7 × 7 grid of square tiles of 20 cm per side on a 5 cm frame. It has walls made of transparent plexiglass. Rats enter this arena from any one of three different start boxes—south (S), east (E) and west (W). An identical but differentially placed box serves as the home box (north). Six cylindrical plexiglass sandwells (6 cm diameter × 6 cm depth, Adam Plastics Ltd.) with a spherical hollow insert (6 cm diameter × 4 cm depth) were used to contain flavoured food as described in Tse et al. (2007). Supreme Mini‐Treats 1‐g flavoured food pellets purchased from Bio‐Serv (item codes are from LBS Serving Biotechnology, United Kingdom) were used: banana (1024041), very berry (1024045), chocolate (1024043), piña colada (1024044) and marshmallow (1024043). In the experiments, these treats were cut in half to produce 0.5‐g pellets. The rewarded plexiglass sandwell was the only one with accessible food. Sandwells were covered in sand (“cage proud” bird sand, Pettex Ltd). Two intramaze objects (a black‐painted 40‐cm water bottle and a 48‐cm stack of glued golf balls) were present in the arena, and several 2D and 3D extra‐maze objects were present in the room. Extramaze cues were about 40–60 cm in size. Sessions were recorded using a ceiling mounted camera (CCTVFirst), positioned centrally above the arena floor and OBS recording software and DeckLink Mini Recorder cards (Blackmagic Design Pty Ltd). The experimenter room and control panel were separated from the arena room by blackout curtains.

### 2.3 Control for Olfactory Cues

**FIGURE 1:**
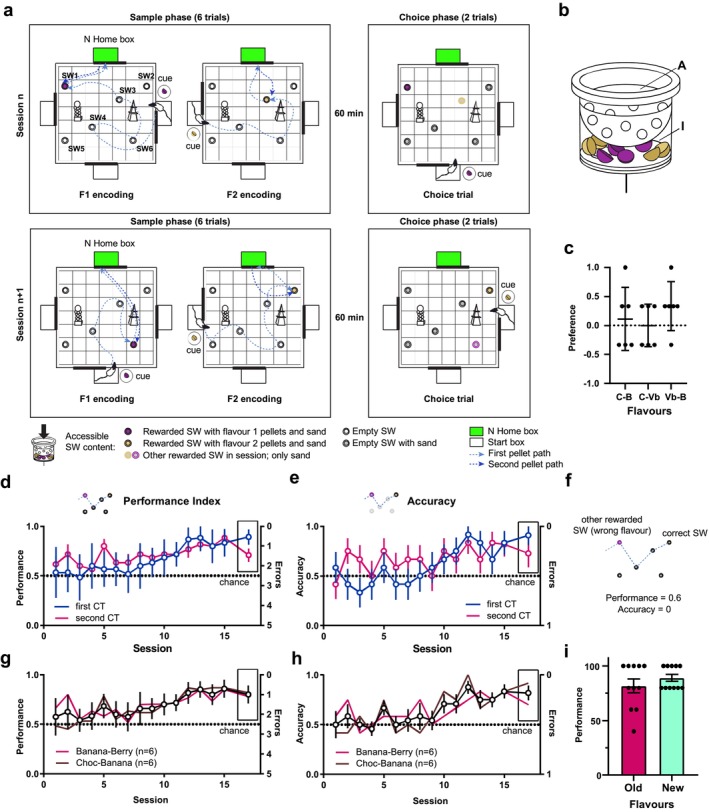
Experiment 1. (a) Schematic of two typical consecutive sessions in Experiment 1. The details of the content of the accessible compartment of the sandwells in the various phases of the session are depicted explicitly with diagrams at the bottom of the panel. (b) Design of the sandwells used in the study, indicating the accessible (A) and inaccessible (I) compartments. (c) Flavour preference for chocolate (C)/banana (B), chocolate (C)/very berry (Vb), and very berry (Vb)/banana (B) combinations. (d) Performance Index (inverse of the number of errors) for various sessions in choice trials (CTs). The Performance Index in the first and second CTs is represented as blue and magenta lines, respectively. Here and in later panels, the boxed session indicates the values in S16 where new flavours where used. (e) Accuracy in the first and second CTs is represented in blue and magenta. (f) Schematic exemplifying the calculation of performance and accuracy for a diagrammatic trajectory. (g) Average Performance Index (CT1‐2) for all rats in the experiment (black line) and values calculated for the two groups using separate flavour combinations (brown‐magenta). (h) Average accuracy (CT1‐2) for all rats in the experiment (black line) and values calculated for the two groups using separate flavour combinations (brown‐magenta). (i) Performance in the last three sessions with old flavours and in the session with new flavours. Throughout the figure, data (bars or empty dots) are presented as mean ± SEM, with black dots representing individual animals.

To maintain uniformity of olfactory cues between the sandwells, all sandwells contained a mixture of pellets of the flavours used in each experiment (five pellets for each flavour) in an inaccessible compartment below the accessible part of the sandwell (Figure 1b). Small (approximately 5 mm in diameter) holes allow the smell to diffuse out while preventing the rats from having access to them. Nonaccessible pellets were refreshed routinely to ensure they maintained a constant smell. Between trials, sandwells were emptied, and their content refreshed. The sandwells were constantly rotated from a pool of approximately 30 plastic sandwells, and the sand was recovered through a sieve to remove pellets, pooled, and mixed for new usage. Fresh sand was added regularly to compensate for sand loss in the procedure. This resulted in the sand having a uniform scent due to the ongoing grinding of food pellets. In Experiment 2, we also run probe trials (see Section 2.7) where no pellet was available in the accessible compartment, and the fraction of time digging at the correct sandwell was measured. This was done in line with previous studies (Nonaka et al. 2017; Gobbo et al. 2022) as a control for olfactory cues.

Between trials and between animals, the arena was cleaned with a 70% ethyl alcohol solution to remove olfactory traces of previous trajectories. At regular intervals, tiles of the arena grid were randomly swapped with tiles from an external pool of floor tiles to further minimise the possibility of animals relying on olfactory traces.

### 2.4 Habituation

To familiarise the animals with food, 10 g of flavoured pellets was left in the rats' cages overnight but buried in sand to encourage digging. Dietary restriction was put in place to maintain animals at 85%–90% of free‐food weight based on a previously established weight curve (Gobbo et al. 2022). During habituation, the rats were individually placed in the open‐field arena to be familiarised with the environment and experimenter handling. Lighting was dimmed to encourage exploration. Animals were encouraged to leave the start box, find the pellets and return to the home box to eat.

### 2.5 Flavour Preference Test

Six rats chosen randomly were used to evaluate their food preference. Two sandwells were open at the same time with the flavours of banana (B), very berry (Vb) or chocolate (C) all accessible. Three trials were run per day for three consecutive days. Two sandwells containing pellets of two different flavours were located equidistant from the start box (S). Rats were placed in the start box, allowed to enter the arena, approach one or the other sandwell with accessible food and choose one of two flavours. Once the subject chose a flavour, they retrieved the food pellet and then entered the N box to eat it, followed by reentering the arena to choose a second pellet, either of the same or a different flavour. This was repeated twice in each session until the food was depleted or 10 min of time had elapsed. A food preference (FP) score was calculated as follows: The two flavours were assigned +1 or −1 values, and for each trial, the corresponding value was assigned based on the flavour of the first pellet chosen by the rat. The FP score is the averaged score across the total number of test sessions.

### 2.6 Experiment 1: What‐Where

The behavioural protocol was similar to Broadbent et al. (2020), with modifications to include two flavours on a daily basis. In each session, the animals learned two novel locations daily for each of the two flavours. For instance, in the example shown in Figure 1a, in session *n*, Flavour 1 was located in SW1, and Flavour 2 was located in SW3; in session *n* + 1, Flavour 1 would be in SW6, and Flavour 2 would be located in SW2, and so on.

For each session, subjects first completed a sample phase of six trials (three for each flavour) where they learned the location of the flavours for that session (Figure 1a). After a time delay of 60 min (50–75 min), the choice phase followed. In the choice phase of each session, one of the two flavours was selected randomly, and the subject's memory was tested to determine whether it could recall the correct location of the flavour cued in the start box. Two trials were run in the choice phase.

Each trial starts from one of three start boxes (W, S, E). Start boxes are black rectangular boxes with a transparent door whose opening and closing can be controlled automatically from the experimenter's computer. At the beginning of each trial, the animal is placed in one of the start boxes with the door closed and receives a cue pellet of the same flavour as that present in the correct sandwell in the arena to instruct it on what flavour to expect. After the cue pellet has been consumed, the door is opened by the experimenter. Once the rat has exited the start box, the door is closed. The experimenter controls the automatic opening and closing of the doors from a part of the experimental room separated by a curtain. The animal can then search for the rewarded well, and, after finding the food reward, the door to the home box is opened, and the rat is instructed to move inside of it. The home box is analogous to a start box in appearance but is located in a stable location (conventionally, N), and rats never start trials from it. After consuming the pellet in the home box, the rat has the opportunity to retrieve a second pellet in the arena and return to N; after entering the home box, the door is closed and the trial ends. Rats quickly learn to navigate autonomously to the home box in a few sessions. Each trial begins from one of the three start boxes (W, S, E) randomly assigned.

In each sample trial (ST), the rewarded location contains three accessible reward pellets of the designated flavour at the bottom of the sand‐filled spherical bowl and more of both flavours in the inaccessible compartment for smell uniformity. The remaining nonrewarded sandwells do not contain sand and have flavoured pellets in the inaccessible compartment only. Six STs are performed alternating between the two flavours. The first flavour was selected randomly. For instance, if the first flavour is Flavour 1, the procedure is as follows: In ST1, a cue pellet of Flavour 1 is given to the rat in the start box (e.g., E start box); then, the rat can enter the arena and learn which sandwell is rewarded with Flavour 1 (e.g., SW1), find pellets of Flavour 1, go to the N home box to consume the pellet, and then retrieve a second pellet from SW1. Following that, ST2 is run, and the rat is put in the designated start box (e.g., W start box), receives a cue pellet of Flavour 2, then learns the location of the sandwell rewarded with Flavour 2 (in the example SW6), goes to N, and then retrieves a second pellet from SW6. STs 3–6 are performed in the same way, alternating between Flavour 1 (STs 3 and5) and Flavour 2 (STs 4 and 6), for a total of six STs per session (three for each flavour).

In the choice phase, the choice of flavour to be identified for each session was determined randomly, with the condition that, in each session, half of the animals were tested on one flavour and half on the other. In choice trials (CTs), all wells appear identical and are filled with sand. All sandwells contain flavoured pellets in the inaccessible compartment (as described above), but only the correct sandwell contains food pellets in the accessible compartment. Choice trials are performed as follows: In CT1, a cue pellet of the designated flavour (e.g., Flavour 2) is given to the rat in the start box (e.g., S start box), then the rat can enter the arena and its performance is tested. Digging or prolonged exploration or sniffing at nonrewarded sandwells is counted as an error. Rats are allowed to perform freely until they dig at the correct sandwell (in the example, SW6) and retrieve the Flavour 2 pellets. At this point, the door of the N home box is opened, and the rat is allowed to enter it to consume the reward and then come back to retrieve a second pellet from SW6. A second CT is then performed identically from the S, W, or E start box, which is randomly selected. Between trials, sandwells are exchanged with fresh ones. Errors are calculated as the number of sandwells in which the animals have been digging in each CT before reaching the correct sandwell (see Section 2.9).

The usage of the start boxes in STs and CTs, the identity of the rewarded sandwells, the identity of the first flavour in the sample phase and the flavour tested in CTs were randomised and counterbalanced between animals using a random generator (https://commentpicker.com/random‐number‐generator.php). The rats were randomly assigned to two groups and trained on alternating days for an initial total of 15 sessions each. Group 1 was trained with Flavour 1 = chocolate and Flavour 2 = banana, whereas for Group 2, Flavour 1 = very‐berry, and Flavour 2 = banana. Then, on Session 16, we used two novel flavours to which the animals had never previously been exposed: Flavour 3 = very‐berry (Vb) and Flavour 4 = marshmallow (MM) for Group 1, and Flavour 3 = chocolate (C) and Flavour 4 = piña colada (PC) for Group 2. Session 16 was run in the same way as earlier, except now with these novel flavours.

### 2.7 Experiment 2: What/Where‐When

#### 2.7.1 Pretraining and Food Preference (S1‐S5)

After completing Experiment 1, the aim was to establish the role of ‘time’ in memory. Procedurally, the animals were given a 2‐week break, while placed on free food. At the beginning of Experiment 2 (E2), they were 6 months old with a mean weight of 479.58 g.

**FIGURE 2:**
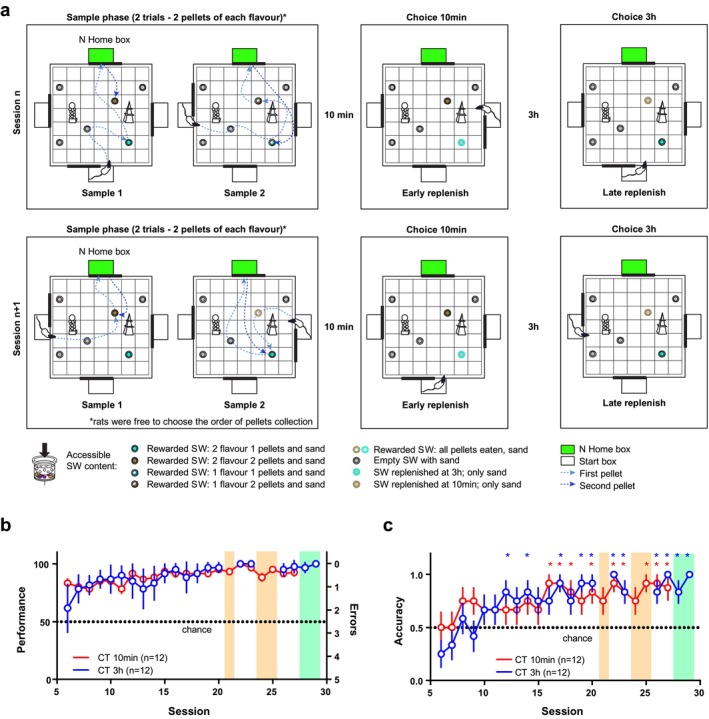
Experiment 2. (a) Schematic of two typical sessions in Experiment 2 as described in the text. In session *n*, we represent an example where the rat collects one pellet for each flavour in the first sample trial (ST). In session *n* + 1, the example shows the case where both pellets for the same flavour are collected in ST1. (b) Performance Index for various sessions in the 10‐min choice trial (red) and 3 h choice trial (blue). (c) Accuracy for the 10‐min choice trial (red) and 3‐h choice trial (blue). Asterisks indicate sessions where the accuracy was significantly above chance according to colour code (*p* < 0.05 Mann–Whitney *U* test, adjusted for multiple comparisons using the false discovery rate with the two‐stage step‐up procedure of Benjamini, Krieger, and Yekutieli; FDR 1.0%). In (b) and (c), orange shading indicates sessions where probe trials were run at 3 h instead of the CT 3 h, whereas green shading indicates sessions where CT 10 min was omitted. Data are presented as mean ± SEM.

In E2, the same arena was used, but the positions of the sandwells (map) were different. Six distinct maps were used for the 12 animals. The sample phase from the above protocol was modified as follows: two flavours (2 × 0.5 g pellets each) from three possible combinations (Vb/MM, C/MM or C/Vb) are accessible at the same time. Rewarded wells each contain two pellets of the same flavour (in the example in Figure 2a, SW3 has two chocolate pellets, and SW6 has two very berry pellets) in the accessible compartment and are covered in sand. They also contain extra pellets of both flavours in the inaccessible compartment to ensure uniform smell. Nonrewarded sandwells contain pellets of both flavours in the inaccessible compartment, but none in the accessible one, and are covered with sand. The positions of the two flavours remain fixed for a given animal across all sessions, whereas the start box usage changes within and between sessions.

For the first five sessions, rats are allowed to form flavour‐place paired associations. Every session, rats complete two STs where they locate both sets of flavours until the STs are depleted. STs were as follows: In ST1, the rat is placed in the start box but receives no cue pellet. When the door opens, it is allowed to explore until one pellet (of any flavour) is retrieved (e.g., Vb at SW6) and then navigate to the N home box to consume it. It then has the chance to return to the arena and retrieve a second pellet. Some rats chose to return to the same sandwell where they had just retrieved a pellet (in the example, SW6), whereas others opted for the other sandwell (SW3). Even the same rats used one or the other option between sessions. The sandwells are then refreshed while maintaining the same number of remaining pellets (e.g., if one pellet of each flavour was taken in ST1, they would contain one pellet each; if both pellets of Flavour 1 were taken, the corresponding rewarded sandwell will only contain sand, and the other rewarded one will still have two pellets of the other flavour in the accessible compartment). ST2 is then performed analogously to ST1, and the rat is only allowed to enter the home box after retrieving a pellet. We have displayed the two types of alternatives in Figure 2a in session *n* and *n* + 1. The examples are nonexhaustive (for instance, rats may start from Flavour 2 instead of Flavour 1).

#### 2.7.2 Training Sessions (S6–S29)

From S6, sessions comprise one sample phase, followed by two CTs. The sample phase is made of two STs as described in the previous paragraph. Rats learn that, after the STs, the two flavours are replenished at two different time intervals. One CT is run after a short (10 min; CT 10 min) and the other after a long (3 h; CT 3 h) time interval (time delay between exposure and recall). Only one of the two flavours is replenished and available in the CT 10 min, whereas the other—and that alone—is replenished after 3 h. The identity of the flavours (i.e., which flavour is replenished at 10 min, and which at 3 h) is randomly assigned to each animal. This identity is consistent across sessions, for example, for a given rat MM is always replenished at 10 min, and Vb at 3 h. Two pellets (0.5 g/pellet) of the available flavour are placed at the bottom of the sandwell in the accessible compartment and buried with sand. The other sandwells are filled with sand; all sandwells have pellets of both flavours in the inaccessible compartment. In both CTs, rats are placed in the designated start box (either 10 min or 3 h), allowed to enter the arena and tested in their search for the correct replenished rewarded well. Once the pellet is found (e.g., MM for CT 10 min), the rat is allowed to carry it to the home box to consume it and then return to the arena to collect a second pellet. Trials end once both pellets have been located and the rat returns to the home box. Sessions were run for a total of 29 days (*n* = 12 rats). Sessions 28 and 29 served as controls to confirm that rats are using the elapsed time as the cue and not simply alternating between flavours. In these sessions, STs were run as usual, but the CT 10 min was omitted, and only CT 3 h was performed 3 h after the end of the ST. Errors are calculated as the number of sandwells where the animals have been digging in each CT before reaching the correct sandwell (see Section 2.9). Data from CTs in Session 27 for rats H5009‐12 (four animals) could not be collected due to circumstances beyond the control of the experimenters; Session 27 therefore represents data for animals H5001‐08.

#### 2.7.3 Probe Trial Sessions (S22, S24 and S25)

To confirm that animals were not using olfactory cues in making choices, probe trial sessions (PT) were conducted during Sessions 22, 24 and 25, as described in Tse et al. (2007), in lieu of the CT at 3 h. In PTs, all six sandwells are identically filled with sand and contain only pellets of both flavours in the inaccessible compartment. Animals were tested on their memory and their focus in digging. In PTs, 3 h after the ST, rats are put in the start box and let into the arena. They are allowed to dig at will for 2 min, at which point a pellet of the flavour to be replenished at 3 h is put in the corresponding sandwell to prevent extinction. The rat is allowed to retrieve it, carry it to the home box and eat it, ending the trial. At the end of the trial, the north door is opened, and, once the animal has entered it, the rat returns to the home box. The relative amount of time spent digging at the various sandwells is calculated during the 2 min PT. Digging time refers to the time during which the animals' forepaws moved the sand with intention to locate pellets beneath. Time spent sniffing, nose‐poking and walking over the sandwell are not considered ‘digging’. The expectation is that the animal remembering the correct location rewarded at 3 h would spend longer digging at this location compared to the other sandwells (see Section 2.9).

### 2.8 Histology

Eight rats were destined for histological confirmation of hippocampal involvement on Session 29. Four rats performed the session as described above (STs and CT 3 h), whereas four rats were only trained in STs and returned to their home cage afterwards. One hour after the CT 3 h or 4 h after STs, rats were perfused for c‐fos staining. Rat was deeply anaesthetised with pentobarbital and perfused with cold phosphate buffered saline (PBS, Merck 524650) and 4% formaldehyde; the brain was fixed overnight in formaldehyde and then immersed in 30% sucrose PBS for 48 h at 4°C. Sixty‐micrometre‐thick coronal sections were cut with a cryostat (~Bregma −3.60 mm). Sections were incubated in 10% normal donkey serum (NDS, Merck 566460), 0.3% Triton X‐100 PBS for 60′ and then overnight with rabbit anti‐c‐Fos IgG (1:1000 dilution, Synaptic Systems 226,308) in PBS with 10% NDS and 0.1% Triton X‐100. Three 5′ washes with PBS were completed before applying the secondary antibody, donkey antiguinea pig Cy3 conjugate (1:200 dilution, Merck AP193C), for 120 min. Slices were washed as before in PBS for 5 min each. The slices were slide‐mounted with Fluoroshield with DAPI (Abcam ab104135) mounting medium. Five‐micrometre z‐stack images were captured on a Nikon Eclipse Ti inverted confocal microscope with 20X objective. After projecting the z‐stack, the same threshold was set for all images and neurons with positive signals were quantified as c‐Fos+/DAPI+ cells with ImageJ CellCounter Plugin (NIH).

### 2.9 Data Analysis

Data were analysed during the experiment and rescored blind by two independent users. Data were analysed with GraphPad Prism (v9.3.1).

#### 2.9.1 Latency

Latency is the time taken from exiting the start box to the start of digging at the correct sand well (seconds).

#### 2.9.2 Number of Errors

The Performance Index (PI) was calculated using the number of sandwells at which the rats dug before locating the correct sandwell, calculated as 100 * (5‐errors) / 5, where chance performance is 50%.

#### 2.9.3 Accuracy

Accuracy is equivalent to the Performance Index calculated considering only the two rewarded wells in the session as possibilities. Accuracy was assigned a value of 1 if rats dug at the correct sandwell or at any other nonrewarded sandwell before that and 0 if they dug at the alternative, incorrect, rewarded sandwell.

#### 2.9.4 Fraction Dig Time and Discrimination Dig Time

In probe sessions, the time spent digging at the correct (or previously rewarded sandwell) is expressed as the fraction of the total digging time at all sandwells (fraction dig time). We also express the total dig time at the correct sandwell (3‐h sandwell) over the total time spent digging at the correct or alternative (10‐min flavour) sandwell (discrimination dig time).

Data are plotted for individual animals in the Supporting Information and as means ± SEM (*n* = 12). Data were analysed using one‐way analysis of variance (ANOVA), mixed linear models or paired sample *t* tests, as appropriate. Performance and accuracy across sessions were assessed by repeated measures two‐way ANOVA with Greenhouse–Geisser corrections and by one‐way ANOVA with Wilcoxon rank‐sum test with a Bonferroni correction as appropriate.

For the immunohistochemical analysis, the mean number of stained nuclei from two sections in the region of interest (dorsal CA1 and DG) was used for group comparisons of c‐Fos expression. Results were analysed using a one‐sample *t* test. Statistical significance was set to *α* = 0.05.

## 3 Results

We sought to determine if the rats were able to form and retrieve independent everyday memories by extending the information tested in the event arena task (Bast et al. 2005; Nonaka et al. 2017). We explicitly incorporated the *what* information in Experiment 1, and *when* in Experiment 2. In the everyday arena paradigm, animals learn to retrieve food pellets from one of multiple sandwell positions in a two‐dimensional arena (Bast et al. 2005). Which sandwell is rewarded each day, instead, constitutes a critical aspect of the everyday memory. In the everyday memory task, there is long‐term memory of the apparatus and the environment, as well as the task outline (Figure 1a). The intensity and the perceived importance of the individual memories learned in each session determine how long they last; this was observed to range from hours to days (Wang et al. 2010). We modelled the *what* aspect of memory with food pellets of different flavours as used in Day et al. (2003) and Tse et al. (2007). The *when* component, instead, was modelled as the time elapsed from food depletion of two flavoured pellets replenished at two different time intervals.

### 3.1 Food Preference

We first determined if rats had any preference towards one of the flavours, which could otherwise bias the search towards the preferred flavour in the following experiments. Rats were placed in a start box and were allowed access to the arena by opening the door remotely (see Section 2.2). Two sandwells were available, each containing three pellets of the same flavour, but the flavours differed between the sandwells. Animals were left to choose which flavour to consume and in which order. Over three trials on different days, there was no significant difference with respect to which flavour was picked first (Flavour Preference Ratio, Figure 1C) (Welch's ANOVA *F*[3, 18] = 1.489 *p* = 0.27) or in the amount eaten (Brown‐Forsythe ANOVA *F*[3, 18] = 1.226 *p* = 0.34) of the two flavours (Figure S1a). We therefore concluded that the flavours chosen were equally appealing to the rats and were used in the following experiments.

### 3.2 Experiment 1: What‐Where

In the everyday memory task, animals learn to retrieve food pellets from one of six possible sandwells. The location of the rewards changes every day (Bast et al. 2005; Wang et al. 2010). Because positions change from day to day, the animals need to remember where they *last* retrieved a certain flavour that day, making this a recency task, hence having an implicit temporal component. To promote the use of an allocentric map, in each trial, they entered the arena from one of three possible start boxes. After entering the arena and retrieving the food pellet from the correct sandwell, they carried it to the home box (conventionally located on the north side of the arena) and then returned to the sandwell for a second pellet (Broadbent et al. 2020).

In Experiment 1, we tested the possibility that rats could form and retrieve two independent memories represented by the positions of food pellets of two different flavours. In the sample trials (STs), they were given the opportunity to learn the flavour–sandwell associations for that session. Rats learned what flavour would be available in each trial by receiving a cue pellet in the start box of the same type. Six STs were performed, three for each flavour. After 60 min., two choice trials (CTs) were performed, where their memory was tested: A cue pellet of the test flavour was given to them in the start box, and the animals had to choose the correct sandwell for that flavour among six identical sandwells (Figure 1a; see Section 2 for full description). To test the ability of animals to freely recall each memory independently from cues, we only tested one of the two flavours in the two CTs, chosen randomly. This way, the second CT only tests the same memory from a different starting point, without incurring in the issue of animals simply searching for the *other* flavour not found in the first CT.

Two groups of animals were trained with different combinations of flavours (either chocolate‐Banana or banana‐very‐berry). To ensure that all sandwells had a similar olfactory profile, they all contained a mixture of the two flavours in the bottom, inaccessible compartment (Figure 1b). Rats in the two groups exhibited similar learning curves (repeated measures REML linear model: performance ~ session * flavour combination + [1|animal]; 1st CT: Session factor *F*[5.39, 57.73] = 2.82 *p* = 0.021; flavour factor *F*[1, 150] = 0.23 *p* = 0.63; average CTs: session factor *F*[4.82, 51.61] = 3.34 *p* = 0.012; flavour factor *F*[1, 150] = 0.61 *p* = 0.44). Their Performance Index (see Section 2) was initially at chance, but it reached above 80% in the last group of sessions (S12–15) (Figures 1d and S1b). Similarly, latency showed a decreasing trend across sessions (REML mixed model latency ~ session + [1|animal], test for linear trend 1st CT: *F*[1, 154] = 7.15 *p* = 0.034) (Figure S1c,d).

A viable way to solve the task would be to dig randomly at one of the two rewarded locations. For this reason, we calculated a measure analogous to the PI by considering only the rewarded wells as the two possible alternatives and termed this parameter Accuracy (see Section 2). Accuracy increased across sessions and was significantly different from chance during sessions 11–15 (Mann Whitney test *p* < 0.05, corrected for multiple measures with the BKY two‐stage step‐up FDR method 0.05; Figure 1e,f). Furthermore, we found no difference in performance and accuracy between trials where the flavour used in the CT was the same or different from the last ST (Figure S1e–h). This rules out that the animals simply alternated between the two rewarded wells (Figure 1d–h; data from individual animals are displayed in Figure S2). One possibility is that the increased performance of the animals is in part due to familiarity towards the flavours used. To exclude this possibility, on Session 16, animals were trained with two novel flavours they had never encountered before (very berry‐marshmallow and chocolate‐piña colada, respectively). The performance with the new flavours was analogous to the PI in the previous three sessions with the old flavours (Student's *t* test = 1.077, df = 10, *p* = 0.3068, Figure 1i). Accuracy was above chance even for the new combination of flavours (*p* = 0.0013, Mann Whitney test corrected for multiple comparisons). This confirms that new information regarding the position and the flavour can be learned and retrieved independently.

### 3.3 Experiment 2: What/Where‐When

In Experiment 2, we sought to model the time component more explicitly, following the paradigm of Clayton and Dickinson (1998). To do this, we designed the experiment so that, after the rats had depleted two flavours in the STs, they would be replenished at different time points from the end of the STs (Figure 2a).

The main task was preceded by five sessions, during which the animals familiarised themselves with two novel flavour combinations and established the *what/where* association (see Section 2). In these five sessions, we also established whether rats had any preference for one of the flavours, without finding evidence in support of any preference (Welch's ANOVA *F*[2, 27.86] = 0.61, *p* = 0.55; Figure S3a). Every session, rats retrieved two pellets for each flavour in the ST, effectively depleting both. This served as a starting point to evaluate the elapsed time. One of the two flavours was replenished after a short interval (10 min), and the other after a long interval (3 h). As shown in Figure 2b, rats progressively learned which flavour was replenished at 10 min, and which at 3 h. The simplest way to solve the task is to simply try one of the two rewarded locations; indeed, this was the strategy initially employed by the animals, and accuracy was not different from chance in the earlier sessions (Figure 2c; data from individual animals are displayed in Figure S4). Progressively, the rats learned which of the two flavours was replenished at 10 min and which at 3 h: Accuracy increased for both short‐ and long‐term replenishment (REML mixed model latency ~ session + [1|animal] test for linear trend; 10 min: *F*[1, 238] = 12.31 *p* = 0.0005; 3 h: *F*[1, 212] = 43.47 *p* < 0.0001) and reliably stayed significantly above chance.

To confirm that rats are using the elapsed time as a cue to remember which flavour is replenished, and not simply alternating between the two, we omitted the CT at 10 min and ran only the CT at 3 h in Sessions 28 and 29. Animals correctly and accurately searched for the flavour replenished at 3 h (Figures 2b,c and S3b). This demonstrates that rats are not learning an order or sequence of flavours to search but explicitly form an association between the identity of the flavour and the time of replenishment.

To control for olfactory cues, in Sessions 21, 24 and 25, probe trials were run where no reward was present in the sandwells in place of CT 3 h. In Probe Trial 1 (PT1, Session 21), rats dug at the two possible rewarded sandwells similarly (repeated measures one‐way ANOVA *F*[1.699, 18.69] = 3.665, *p* = 0.052). It is not uncommon in this type of task for animals to be confused by not finding the expected reward in the first probe trial, therefore opting to try other sandwells (Gobbo et al. 2022; Tse et al. 2023). Indeed, accuracy in PT1 was essentially at chance, and the low relative fraction time was due to continued digging at the wrong sandwell (Figure S3d,e). When we repeated probe trials in place of CT 3 h in Sessions 24 and 25 (PT2 and PT3), animals dug significantly more at the expected sandwell, that is, the sandwell expected to be replenished at 3 h (repeated measures one‐way ANOVA PT2: *F*[1.180, 12.98] = 17.74 *p* = 0.0007, PT3: *F*[1.191, 13.11] = 20.92 *p* = 0.0003; Figure S3c–e). This confirmed that rats correctly searched for the flavour rewarded at 3 h and excluded the use of olfactory cues to retrieve it. Furthermore, it demonstrates that rats correctly discriminated between the flavour replenished at 3 h and the one replenished at 10 min (Figure S3e).

### 3.4 Hippocampal Recruitment

**FIGURE 3:**
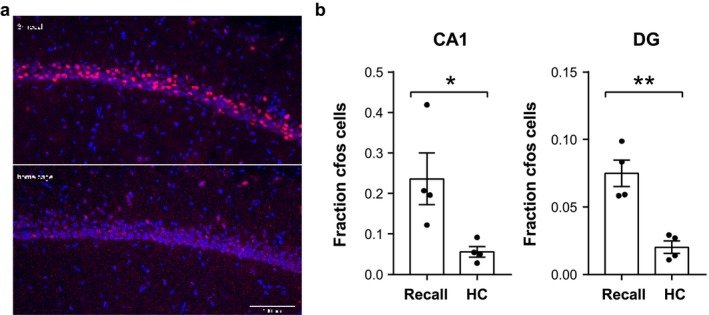
Experiment 2. (a) Representative images of the CA1 area from 3‐h recall and home cage animals stained for DAPI (blue) and cfos (red). (b) Quantification of the fraction of cfos+ cells in CA1 or DG. Bars indicate mean ± SEM, with dots representing individual animals. *p < 0.05, **p < 0.01 Student's *t* test.

To confirm that the hippocampus was involved in the solution of the task in Experiment 2, on S29, we sacrificed four rats 60 min after performing long‐term retrieval (CT 3 h), as well as four other animals that were trained in the ST but were excluded from the CTs. Instead of being tested in the CT 3 h, these four animals remained in their home cage for 4 h, matching the two groups. In animals performing the CT 3‐h trial, a significantly higher number of cells expressed c‐fos in the upper DG blade and CA1 pyramidal layer, which contained virtually only the cell bodies of excitatory neurons (Figure 3). This confirmed that recalling the identity of the flavour replenished at 3 h involved hippocampal activity.

## 4 Discussion

Episodic memories have a central role in everybody's life by shaping our experience and forming our identity. They combine information about the event itself (*what*), the location (*where*)—or sometimes the context surrounding it—and the time where it happened (*when*) (Tulving 1985). The majority of available evidence points to the hippocampal formation and the medial temporal lobe in general as key brain areas dedicated to their processing (Nyberg et al. 1996; Nadel and Moscovitcht 1997; McKenzie et al. 2024). They are severely affected by neurodegenerative disease such as Alzheimer's disease (Knopman et al. 2021). In the last decades, our understanding of information processed by memory areas has advanced greatly, but we need refined models to study advanced brain functions. The theoretical discussion of whether animals possess an equivalent form of episodic memory—which has led to the definition of the concept “episodic‐like” memory for noetic reasons (Tulving 2005)—has found compelling evidence in favour of it as a concept (Clayton and Dickinson 1998; Clayton et al. 2001; Eichenbaum et al. 2005; Babb and Crystal 2006a).

### 4.1 Opportunities and Limitations of Previous Models

Reliable behavioural tasks to model episodic‐like memories are notoriously hard to implement. Tasks such as novel object recognition, object‐place memory and object‐place‐context memory have—unfortunately—been found to be based on familiarity rather than explicit recall (Ennaceur et al. 1997; Wilkinson et al. 2006). Object‐place‐context discrimination, but not object‐place, was found to be sensitive to fornix lesions extending to the hippocampus (Eacott and Norman 2004). More recently, episodic‐like aspects such as *what‐where* or *what‐when* associations have been modelled using the framework of the E maze (Eacott et al. 2005). These have also been investigated by baiting different arms of the radial arm maze with food items of different flavours (Babb and Crystal 2006a; Naqshbandi et al. 2007; Zhou and Crystal 2009). Although Easton et al. (2009) demonstrated that fornix/hippocampal lesions impair recollection but not familiarity, the involvement of the hippocampus in tasks such as those used by Babb and Crystal (2006a), however, has not yet been investigated. This is an important aspect, as surprisingly many tasks can be solved in even the absence of hippocampal activity, likely due to the existence of multiple parallel memory and navigation systems such as the striatum (Packard and McGaugh 1996; Langston and Wood 2010; Broadbent et al. 2020; Duszkiewicz et al. 2023). The general rule seems to be that hippocampal involvement tends to decrease with the complexity of the task or its solvability with algorithmic or stereotypical steps.

The event arena is similar to the water maze in that it is a ‘memory recall’ task (Nonaka et al. 2017). Animals enter the arena from separate start boxes, thereby disentangling recall from the overt execution of the task following a decision (Gobbo et al. 2022). Compared to the water maze, it has the advantage of being a dry maze, hence enabling electrophysiological or optical recordings, and it is an appetitive, or positive reinforcement task, making it a preferable choice in terms of refinement. The disadvantage is that unless opportunely designed, tasks can be ambiguously solved with easier, less computationally demanding approaches that do not necessarily rely on the hippocampus (Broadbent et al. 2020). Furthermore, to date, the event arena has been used to model spatial memory, without explicitly including temporal or item information. Previous attempts to model *what* information with individual flavours were not conclusive as no appropriate analyses and controls were included (Day et al. 2003).

Realistically, animal experiments aim to model some, but not all aspects of episodic or everyday memories, especially when the sampling of numerous trials is necessary to define neural fields and responses using statistical inference. Here, we have built on the allocentric version of the event arena task to (i) incorporate *what*, *where*, *when* aspects of everyday memories and (ii) separate task execution and planning, thus allowing the identification of neural correlates of memory‐based decision making. Although scrub‐jays experiments would offer, in principle, a desirable framework, birds are evolutionarily more distant than mammals to humans; nevertheless, the first attempts at recording neural activity during caching have been recently pioneered and could offer new insight into the processing of multimodal information in episodic‐like memories (Chettih et al. 2024). Here, we have established two protocols that enable the explicit study of *what* and *when* in memory recall studies in rats. Future work may build on this to explicitly model the three aspects in the same behavioural task.

### 4.2 Advantages and Limitations of Experiment 1

In Experiment 1, we demonstrate that rats are capable of learning the location of two independent flavour‐sandwell (*what‐where*) combinations in a daily manner. The ability to perform above chance even with new flavours never previously encountered demonstrates that the animals rapidly form a well‐rounded memory of finding a particular food in a defined location. Still, a number of limitations can be identified.

First, although both performance and accuracy rise above chance in later sessions, the learning curve is somewhat flat for the first 10 sessions. In contrast, rats typically learn the single‐flavoured task in about five sessions or less when using an allocentric protocol (Broadbent et al. 2020; Gobbo et al. 2022). This raises the possibility that rats may have difficulties in understanding the two main rules necessary to identify the correct sandwell when two sandwells have to be learned together: (i) that the position of the correct sandwell is the same regardless of the starting box (allocentric rule) and (ii) that the flavour cued in the start box will be present in the arena (*what* rule). One possibility would be to train rats in the allocentric protocol initially with one flavour (or nonflavoured pellets) and introduce multiple flavours once the choice rule has been learned. Second, although we randomised the flavour identity in the first ST, we alternated the two flavours in STs 1–6 (see Section 2). This might create an expectation in the animals for flavours to be alternated; an improvement of the protocol would be to randomise the order of flavours across the six STs. Third, the use of time in Experiment 1 is only implicit. Rats learn to retrieve food found at a given sandwell during that session, not previous ones. Further work may build on this framework to incorporate an explicit temporal component. Fourth, a property of many instances of episodic memories in humans is the single‐event or single‐trial learning. In Experiment 1, rats experience each flavour in three trials; we believe this was a necessary compromise for them to form a reliable representation, given the complexity of the task, and to promote allocentric behaviour. For this reason, we underline the use of ‘everyday memory’ instead of ‘episodic‐like’ to highlight this point. A possible extension of the protocol would be the introduction of single‐trial learning sessions once rats have reached a steady performance with three trials. This approach has been employed, for instance, in Babb and Crystal (2006a), where tests of episodic‐like memory are preceded by several sessions with multiple rewarded trials per day (up to five visits to arms with nonchow rewards—grape and raspberry), reflecting the need of animals to not only remember associations but also to learn the rules of the task. Furthermore, in humans as well as animals, it has been found that repetition is beneficial for the formation of memories and recall performance typically correlates with the number of repetitions (Kahana 2012).

### 4.3 What/Where Overlap in Experiment 2

In Experiment 2, we show that rats can learn that individual flavours in different locations are replenished at different times. Probe trials and tests run at 3 h in the absence of 10 min tests (Figure 2) are appropriate controls for demonstrating that rats use the absolute time elapsed from the ST to decide which flavour to retrieve, rather than alternating between the two. However, in the design of the task, we introduced a fixed association between each flavour and its location in the experiment. Hence, it remains to be addressed to what extent the *what* component is represented in the neural network of animals solving the task. Although both *what* and *where* components may be represented at the neuronal level, the task can be solved in principle by only remembering which of the two locations is replenished at a given interval. The task therefore models *where* and *when* explicitly. To completely mirror Clayton and Dickinson's (1998) experiments in scrub jays, one could extend the task by shifting the position of the two flavours daily or, at least, regularly. One problem we might envision in doing that is the difficulty for animals to learn the rules of such a task *de novo*. In scrub jays, this was facilitated by their preference for worms compared to peanuts, as well as their natural pilfering habit. One possibility would be to first train rats as in Experiment 2 and when they have reached sufficient accuracy (in our case, approximately 15 sessions), begin shifting the positions of the two flavours between sessions, similarly to what happens in Experiment 1.

### 4.4 Nature of Time in Experiment 2

Experiment 2 used the replenishment time for animals to decide which flavour they should search for. The time used in the experiment is relative time elapsed from food depletion in STs, similar to experiments performed by Babb and Crystal (2006a, 2006, b) and Naqshbandi et al. (2007), as well as Clayton and Dickinson (1998). This is different from explicit time in the form of a calendar, or in the case of animals, circadian time. Although there have been claims that rats can use the latter (Zhou and Crystal 2009), these have not been supported by evidence from other experiments (Roberts et al. 2008). Humans themselves remember poorly the dates, or order, of events in their memories (Friedman 1993). Typically, the temporal information is inferred, or reconstructed, from contextual information (e.g., while I was in college), or when events have a natural serial order (e.g., the sequence of events in a robbery). Hence, as also in Gaffan (1994), the temporal information in episodic memory can be expressed in a contextual way as ‘the occasion in which’ (Easton and Eacott 2008; Prescott et al. 2019; McKenzie et al. 2024). Incidentally, elapsed time might be a more useful feature to remember in practical terms and indeed time cells seem to map the time since a salient event (MacDonald et al. 2011).

In Experiment 2, the increase in the accuracy curve in sessions omitting the choice trial at 10 min and probe trials at 3 h demonstrates that rats use elapsed time as a judgement to search for the replenished flavour. We chose to perform these controls at the end of the learning curve, but future experiments may perform these controls at various learning stages to provide additional information. Furthermore, PTs at 10 min ought to be performed to further support the interpretation of the data. In Experiment 2, we tested two different elapsed time delays (10 min and 3 h). The aforementioned tests suggest that the likeliest use of time is an evaluation of elapsed time (short or long). In scrub jay experiments, this is further supported by the use of two preferred food types with distinct decay times (28 h for mealworms and 100 h for crickets; Clayton et al. 2001). Our design with two time points does not exclude completely that time delay is used in a contextual way. As discussed, both types of time can be used in human memories; a further improvement of the behavioural protocol of Experiment 2 could use three flavours/locations and test them at different time points. An alternative approach taken by Easton and Eacott (2010) models *when* as pure *which* contextual information; an extension of this approach using the event arena would be very similar to what was previously reported in Prodan et al. (2022).

In conclusion, the experiments described here provide new paradigms for future studies to investigate how the various aspects of everyday memories are stored and retrieved during decision‐making (Gobbo et al. 2022). Future work will elucidate if these protocols can be extended to incorporate additional aspects of episodic‐like memories such as single‐trial learning. Hopefully, these will be useful in the design of experiments to advance our understanding of memories and how they guide complex information processing when making decisions based on past experiences.

## Author Contributions

**Kayleigh Kanakis:** formal analysis, investigation, methodology, writing – review and editing. **Richard G. M. Morris:** funding acquisition, supervision, writing – review and editing. **Francesco Gobbo:** conceptualization, data curation, formal analysis, investigation, methodology, supervision, visualization, writing – original draft, writing – review and editing.

## Conflicts of Interest

The authors declare no conflicts of interest.

## Peer Review

The peer review history for this article is available at https://www.webofscience.com/api/gateway/wos/peer‐review/10.1111/ejn.70278.

## Supporting information


**Figure S1:** Experiment 1. (**a**) Amount of food eaten for the various flavours in the food preference test. (**b**) First CT Performance Index. Colour lines indicate the performance in the two groups of animals. (**c**) Average Latency (time to reach the correct sandwell) for Experiment 1 in seconds. (**d**) Latency for Choice trials 1 (black) and 2 (grey). Boxed session indicates the Performance in S16 where new flavours where used. Data (bars or empty dots) are presented as mean±SEM, with small black dots representing individual animals. (**e**) Performance index in the first Choice trial divided by type of flavour: blue dots represent trials where the flavour in the first Choice was the same flavour used in the last (sixth) Sample trial, cyan dots are trials where the flavour in the first Choice was different from the flavour used in the last (sixth) Sample trial. (**f**) Same as in (**e**) for Accuracy. Plots are mean±SEM Numbers are animals. (**g**) Average Performance, respectively, between sessions 10 and 15. Paired t‐test t = −0.72 *p* = 0.48 (**h**) Average Accuracy, respectively, between sessions 10 and 15. Paired t‐test t = −0.69 *p* = 0.51 Bars are mean±SEM and dots individual animals.
**Figure S2:** Experiment 1. (**a**) Performance Index for single animals in Experiment 1. (**b**) Accuracy for individual animals in Experiment 1. Blue and magenta lines indicate the first and second CT, respectively. In Purple, the average value for each animal.
**Figure S3:** Experiment 2. (**a**) Flavour preference for Chocolate (C)/Marshmallow (MM), Chocolate (C)/Very berry (Vb) and Very berry (Vb)/Marshmallow (MM) combinations. (**b**) Performance in the 3 h Choice trial when following a 10 min Choice trial (S27) or not (S28). (**c**) Fraction of digging time spent on the correct (flavour replenished at 3 h), alternative (replenished at 10 min), or incorrect sandwells. PT1–3: probe trials 1, 2, and 3. (**d**) Accuracy calculated for PT1–3 based on the order of visits to sandwells during probe trials. (**e**) Discrimination expressed as the fraction of digging time considering only the time spent digging at the correct or alternative sandwell (i.e., excluding incorrect sandwells). Data (bars or dots in **d**) are presented as mean±SEM, with filled dots representing individual animals.
**Figure S4:** Experiment 2. (**a**) Performance Index for single animals in Experiment 2. (**b**) Accuracy for individual animals in Experiment 2. Red and blue lines indicate the CT 10 min and CT 3 h, respectively.

## Data Availability

Data from this publication can be accessed at the University of Edinburgh DataShare (https://doi.org/10.7488/ds/7910). Original videos, images and records are available upon request from the corresponding author.
