# Supplementary material for: Modelling of What‐Where‐When Everyday Memories in Rats
Source: Eur J Neurosci. 2025 Oct 14;62(7):e70278. doi: 10.1111/ejn.70278 (PMC12519926; doi:10.1111/ejn.70278)
Supplement: Supplementary file 1 — Figure S1: Experiment 1. (a) Amount of food eaten for the various flavours in the food preference test. (b) First CT Performance Index. Colour lines indicate the performance in the two groups of animals. (c) Average Latency (time to reach the correct sandwell) for Experiment 1 in seconds. (d) Latency for Choice trials 1 (black) and 2 (grey). Boxed session indicates the Performance in S16 where new flavours where used. Data (bars or empty dots) are presented as mean±SEM, with small black dots representing individual animals. (e) Performance index in the first Choice trial divided by type of flavour: blue dots represent trials where the flavour in the first Choice was the same flavour used in the last (sixth) Sample trial, cyan dots are trials where the flavour in the first Choice was different from the flavour used in the last (sixth) Sample trial. (f) Same as in (e) for Accuracy. Plots are mean±SEM Numbers are animals. (g) Average Performance, respectively, between sessions 10 and 15. Paired t‐test t = −0.72 p = 0.48 (h) Average Accuracy, respectively, between sessions 10 and 15. Paired t‐test t = −0.69 p = 0.51 Bars are mean±SEM and dots individual animals. Figure S2: Experiment 1. (a) Performance Index for single animals in Experiment 1. (b) Accuracy for individual animals in Experiment 1. Blue and magenta lines indicate the first and second CT, respectively. In Purple, the average value for each animal. Figure S3: Experiment 2. (a) Flavour preference for Chocolate (C)/Marshmallow (MM), Chocolate (C)/Very berry (Vb) and Very berry (Vb)/Marshmallow (MM) combinations. (b) Performance in the 3 h Choice trial when following a 10 min Choice trial (S27) or not (S28). (c) Fraction of digging time spent on the correct (flavour replenished at 3 h), alternative (replenished at 10 min), or incorrect sandwells. PT1–3: probe trials 1, 2, and 3. (d) Accuracy calculated for PT1–3 based on the order of visits to sandwells during probe trials. (e) Discrimination expressed as the f [file EJN-62-0-s001.pdf]

Modelling of what-where-when everyday memories in rats

Kayleigh Kanakis[1], Richard GM Morris[2], Francesco Gobbo[2,3*]

[1]School of Psychology and Neuroscience, College of Medical, Veterinary, and Life Sciences, University of Glasgow, G12 8QQ, Glasgow (UK)

[2]Centre for Discovery Brain Sciences, the University of Edinburgh, 1 George Square, EH8 9JZ, Edinburgh (UK)

[3]UK Dementia Research Institute, the University of Edinburgh, 1 George Square, EH8 9JZ, Edinburgh (UK)

Correspondence: fgobbo@ed.ac.uk

Supplementary Material

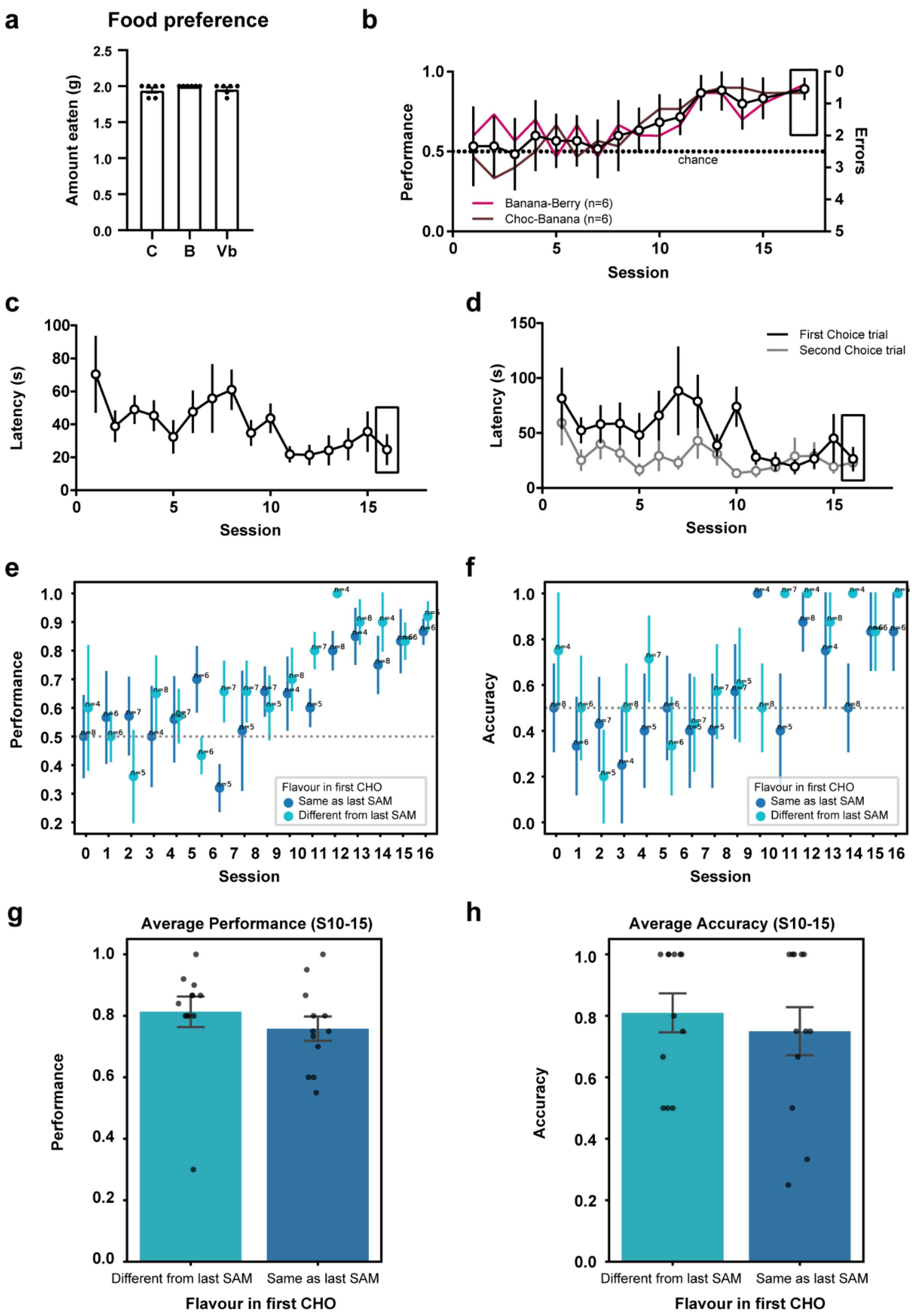

a
Food preference
Amount eaten (g)
C
B
Vb
b
Performance
Errors
chance
Banana-Berry (n=6)
Choc-Banana (n=6)
Session
c
Latency (s)
Session
d
Latency (s)
First Choice trial
Second Choice trial
Session
e
Performance
Flavour in first CHO
Same as last SAM
Different from last SAM
Session
f
Accuracy
Flavour in first CHO
Same as last SAM
Different from last SAM
Session
g
Average Performance (S10-15)
Performance
Different from last SAM
Same as last SAM
Flavour in first CHO
h
Average Accuracy (S10-15)
Accuracy
Different from last SAM
Same as last SAM
Flavour in first CHO

Supplementary Figure S1 - Experiment 1. (**a**) Amount of food eaten for the various flavours in the food preference test. (**b**) First CT Performance Index. Colour lines indicate the performance in the two groups of animals. (**c**) Average Latency (time to reach the correct sandwell) for Experiment 1 in seconds. (**d**) Latency for Choice trials 1 (black) and 2 (grey). Boxed session indicates the Performance in S16 where new flavours where used. Data (bars or empty dots) are presented as mean±SEM, with small black dots representing individual animals. (**e**) Performance index in the first Choice trial divided by type of flavour: blue dots represent trials where the flavour in the first Choice was the same flavour used in the last (sixth) Sample trial, cyan dots are trials where the flavour in the first Choice was different from the flavour used in the last (sixth) Sample trial. (**f**) Same as in (**e**) for Accuracy. Plots are mean±sem. Numbers are animals. (**g**) Average Performance, respectively, between sessions 10 and 15. Paired t-test $t=-0.72$ $P=0.48$ (**h**) Average Accuracy, respectively, between sessions 10 and 15. Paired t-test $t=-0.69$ $P=0.51$ Bars are mean±sem and dots individual animals.

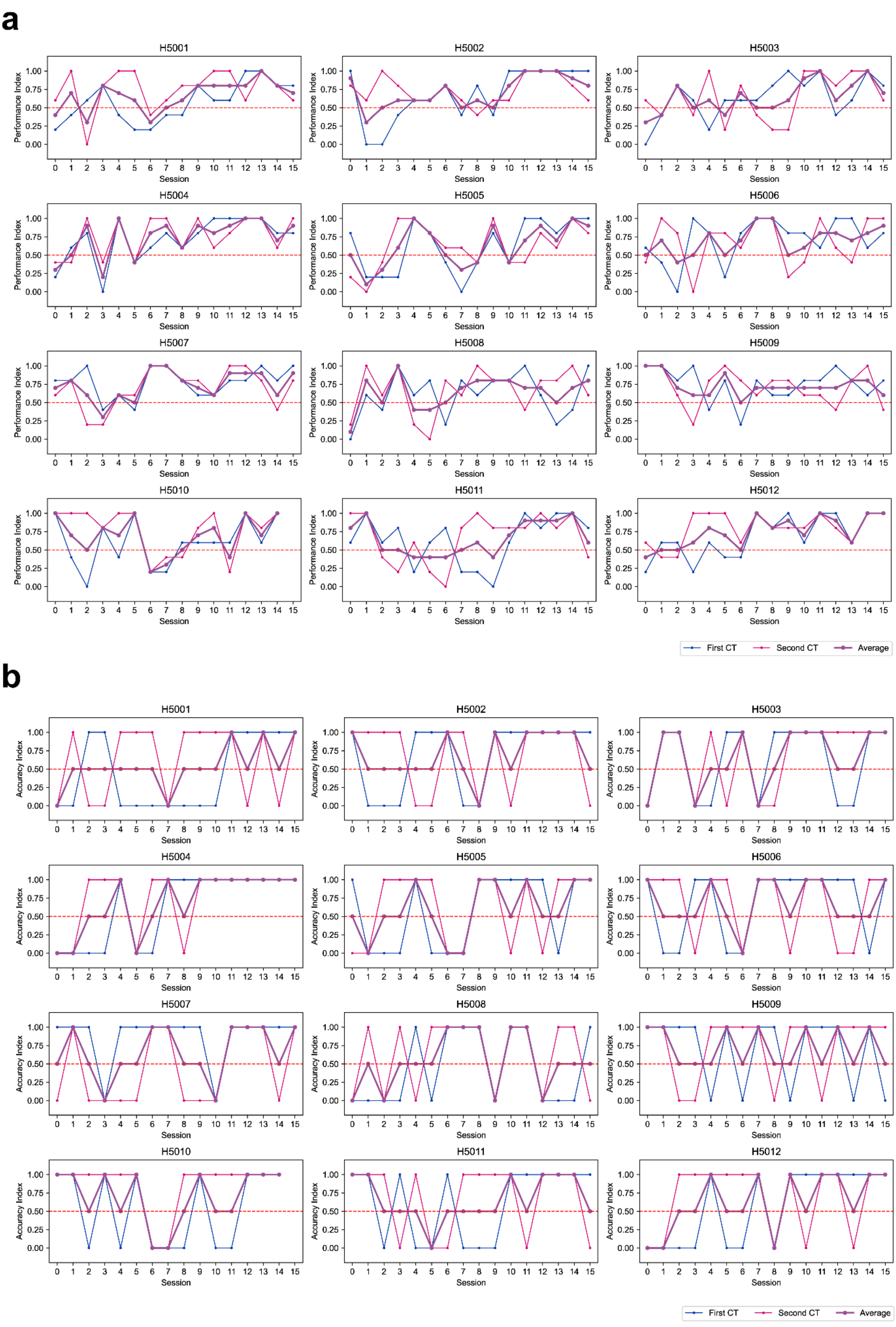
a
H5001
H5002
H5003
H5004
H5005
H5006
H5007
H5008
H5009
H5010
H5011
H5012
Performance Index
Session
First CT
Second CT
Average
b
H5001
H5002
H5003
H5004
H5005
H5006
H5007
H5008
H5009
H5010
H5011
H5012
Accuracy Index
Session
First CT
Second CT
Average

Supplementary Figure S2 - Experiment 1. (**a**) Performance Index for single animals in Experiment 1. (**b**) Accuracy for individual animals in Experiment 1. Blue and magenta lines indicate the first and second CT, respectively. In Purple, the average value for each animal.

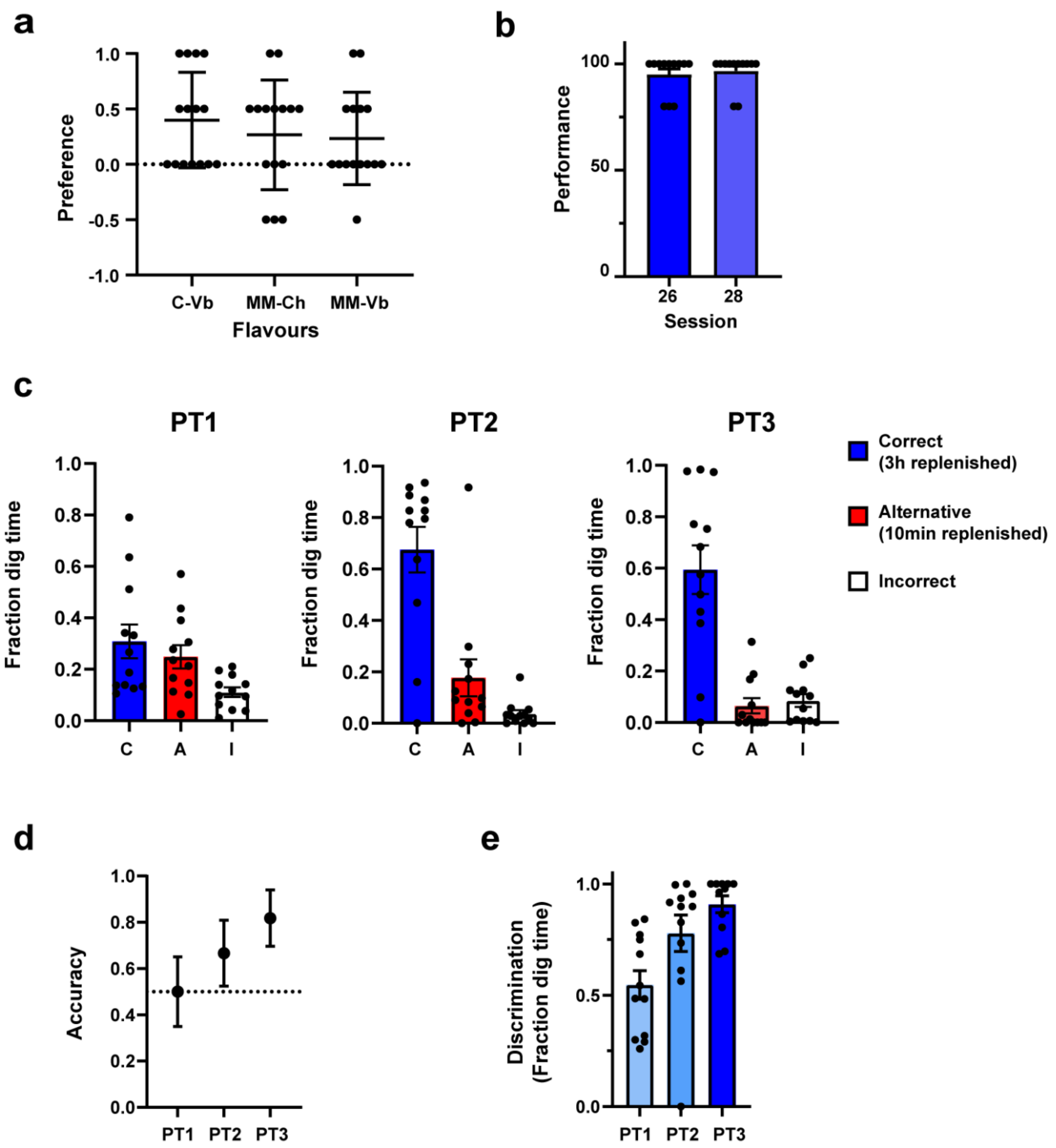

Supplementary Figure S3 - Experiment 2. (**a**) Flavour preference for Chocolate(C)/Marshmallow (MM), Chocolate (C)/Very berry (Vb) and Very berry (Vb)/Marshmallow (MM) combinations. (**b**) Performance in the 3 h Choice trial when following a 10 min Choice trial (S27) or not (S28). (**c**) Fraction of digging time spent on the correct (flavour replenished at 3 h), alternative (replenished at 10 min), or incorrect sandwells. PT1-3: probe trials 1, 2, and 3. (**d**) Accuracy calculated for

PT1-3 based on the order of visits to sandwells during probe trials. (**e**) Discrimination expressed as the fraction of digging time considering only the time spent digging at the correct or alternative sandwell (i.e. excluding incorrect sandwells). Data (bars or dots in **d**) are presented as mean±SEM, with filled dots representing individual animals.

a

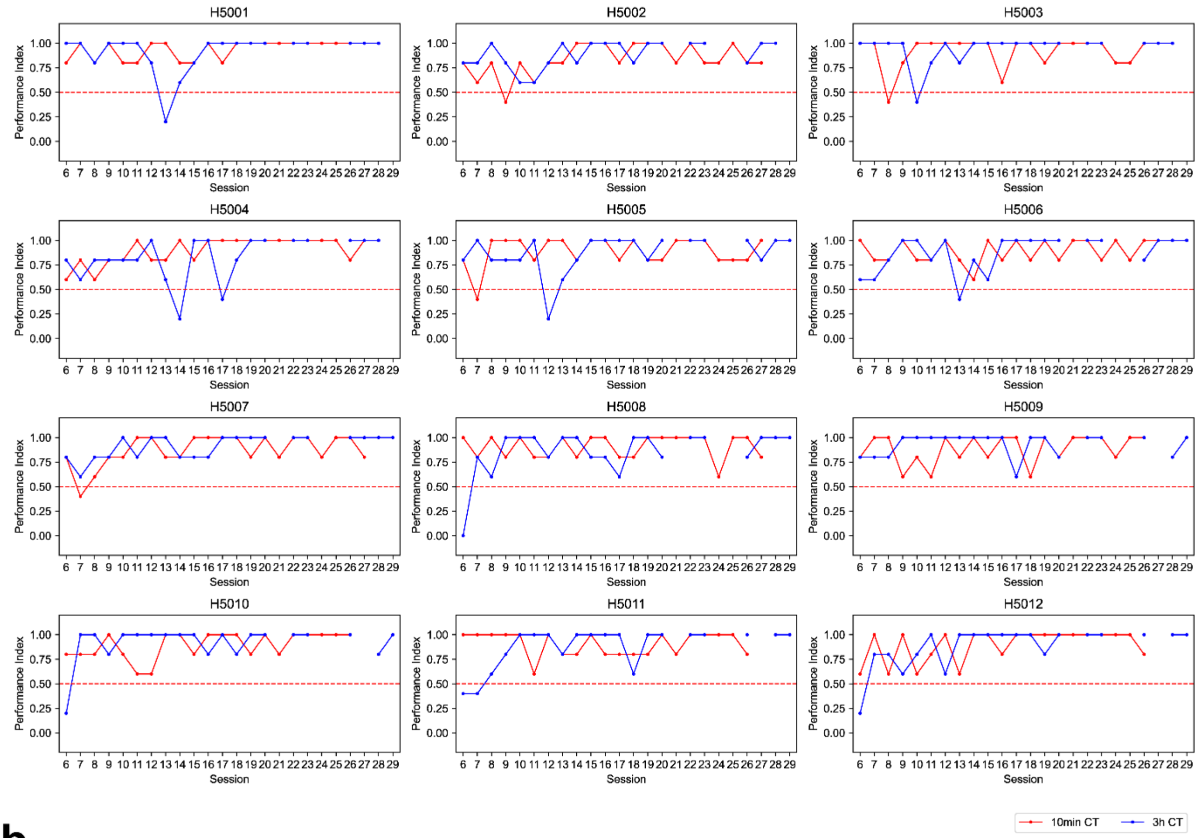
H5001
H5002
H5003
H5004
H5005
H5006
H5007
H5008
H5009
H5010
H5011
H5012
Performance Index
Session
10min CT
3h CT

b

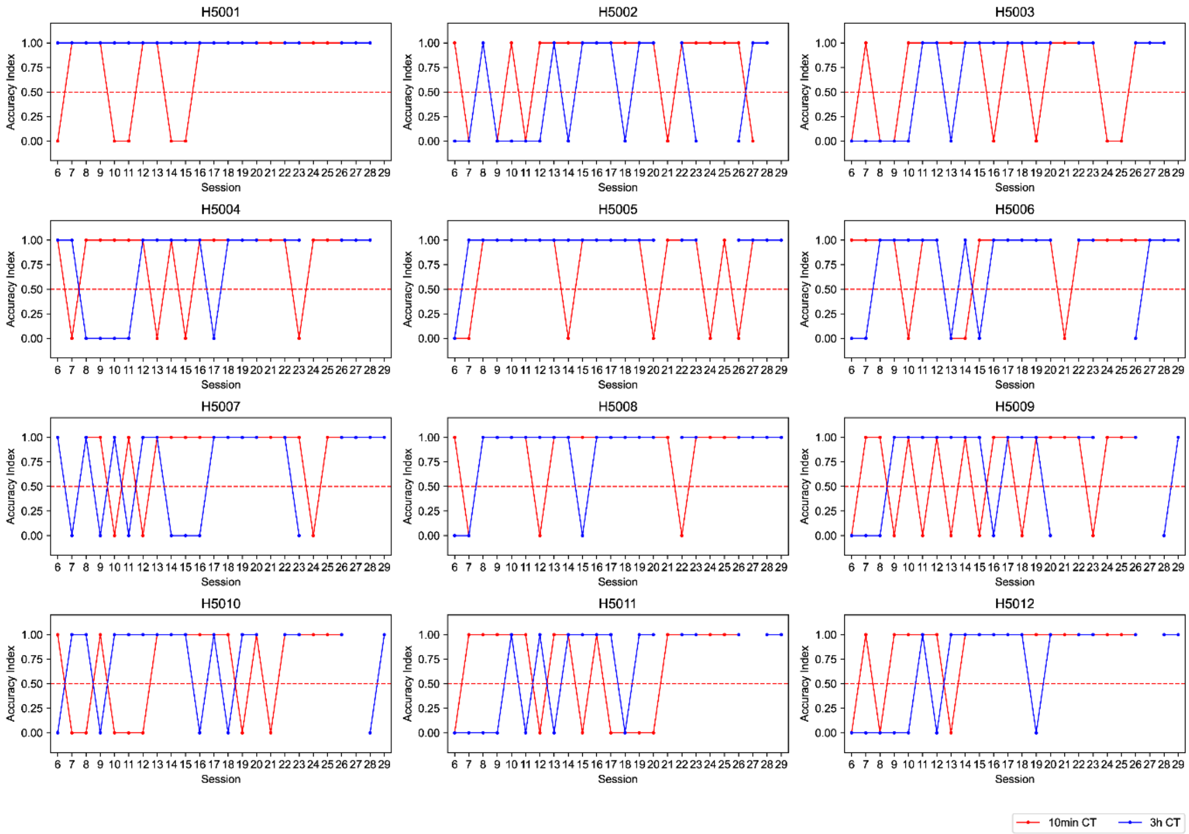
H5001
H5002
H5003
H5004
H5005
H5006
H5007
H5008
H5009
H5010
H5011
H5012
Accuracy Index
Session
10min CT
3h CT

Supplementary Figure S4 - Experiment 2. (**a**) Performance Index for single animals in Experiment 2. (**b**) Accuracy for individual animals in Experiment 2. Red and blue lines indicate the CT 10 min and CT 3h, respectively.
